# Dissecting the regulation and function of ATP at the single-cell level

**DOI:** 10.1371/journal.pbio.3000095

**Published:** 2018-12-14

**Authors:** Jianhan Zhang, Xu Han, Yihan Lin

**Affiliations:** 1 Center for Quantitative Biology and Peking-Tsinghua Joint Center for Life Sciences, Academy for Advanced Interdisciplinary Studies, Peking University, Beijing, China; 2 The MOE Key Laboratory of Cell Proliferation and Differentiation, School of Life Sciences, Peking University, Beijing, China

## Abstract

Regulation of cellular ATP level is critical for diverse biological processes and may be defective in diseases such as cancer and mitochondrial disorders. While mitochondria play critical roles in ATP level regulation, we still lack a systematic and quantitative picture of how individual mitochondrial-related genes contribute to cellular ATP level and how dysregulated ATP levels may affect downstream cellular processes. Advances in genetically encoded ATP biosensors have provided new opportunities for addressing these issues. ATP biosensors allow researchers to quantify the changes of ATP levels in real time at the single-cell level and characterize corresponding effects at the cellular, tissue, and organismal level. Along this direction, several recent single-cell studies using ATP biosensors, including the work by Mendelsohn and colleagues, have started to uncover the principles for how genetic and nongenetic parameters may modulate ATP levels to affect cellular functions and human health.

## Abstract 2

This Primer explores how a systematic understanding of cellular ATP regulation and function at the single-cell level is now becoming possible with the help of CRISPRi-based screening and genetically-encoded ATP biosensors.

## Mitochondria: ATP production and beyond

**Fig 1:**
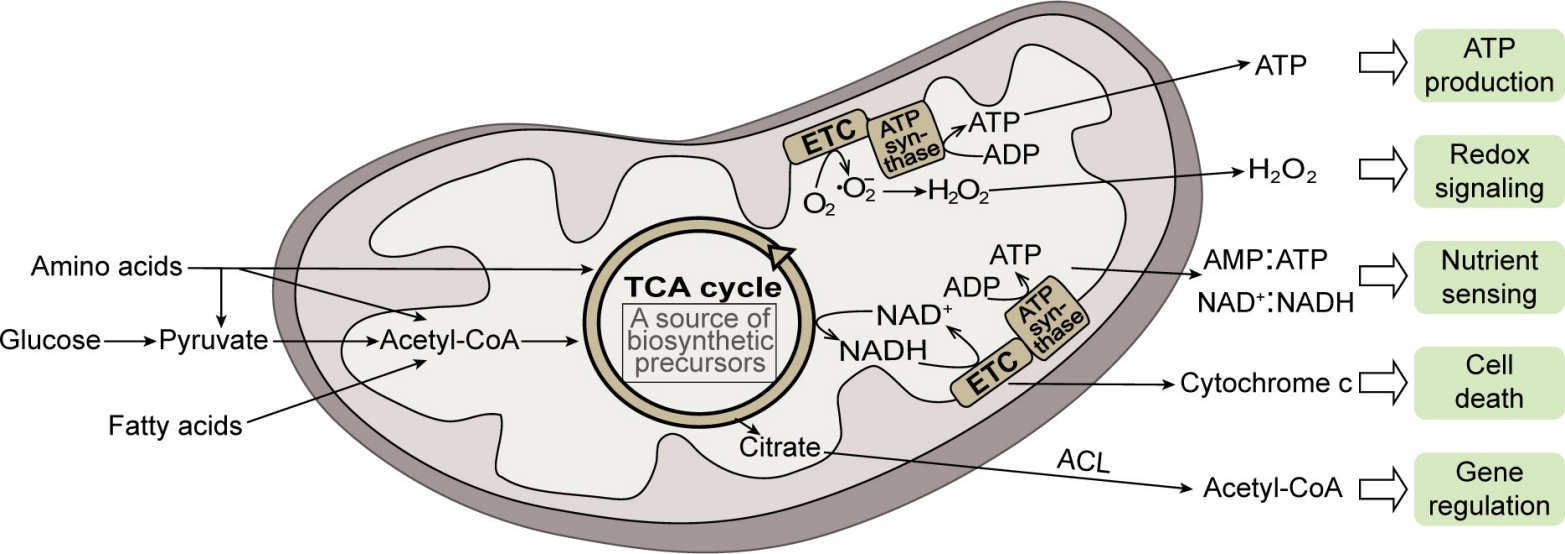
The mitochondrion is a multifunctional organelle. During mitochondrial bioenergetics, acetyl-CoA converted from food molecules is oxidized to produce ATP. In addition to ATP production, mitochondria play essential roles in cell signaling including nutrient sensing, redox signaling, cell death, and many other functions [3, 7]. Note that biosynthetic pathways in mitochondria [4] are omitted in the diagram. ADP, adenosine diphosphate; AMP, adenosine monophosphate; CoA, coenzyme A; ETC, electron transport chain; TCA, tricarboxylic acid.

Mitochondria originated from an ancient alpha-proteobacterium engulfed by a eukaryote ancestor and have coevolved with the host for a long period of time [1]. Mitochondria have acquired many functions through this long history of evolution and participate in numerous biological processes [2]. Mitochondria are best known as the powerhouses of the cell that produce ATP via oxidative phosphorylation (OXPHOS). Mitochondrial ATP synthesis involves tricarboxylic acid (TCA) cycle enzymes, electron transport chain complexes, and ATP synthase, in which acetyl-coenzyme A (CoA) derived from food molecules is oxidized to produce ATP. During bioenergetic reactions, mitochondria also produce other physiologically important molecules such as reactive oxygen species [3] (**Fig 1**). In addition, the biosynthetic function of mitochondria has far-reaching implications in stress responses and diseases [4–6]. Along these lines, increasing evidence shows that mitochondria play essential roles in cell signaling pathways, gene regulation, cell death, and many other functions [2, 3, 7] (**Fig 1**). Understanding how mitochondrial dysfunctions contribute to human diseases is thus challenging and requires a systems-level knowledge of mitochondrial biology.

In mammals, in addition to the 13 proteins produced in mitochondria, more than 1,000 proteins encoded in the nucleus and synthesized in the cytosol are targeted to mitochondria [8]. Mutations in mitochondrial-related genes can disrupt normal mitochondrial gene expression regulation and other functions and are considered as common causes of mitochondrial disorders and diseases [5, 6, 9]. To understand how genotypes are linked to mitochondrial-related disease phenotypes, researchers often need to characterize a set of parameters of the diseased mitochondria [10]. Among these parameters, ATP production level is imminently related to energy failure resulting from mitochondrial dysfunctions. Energy failure may underlie many inherited and degenerative diseases [11] and can manifest in a wide range of ages and organs [9].

Understanding the causes and consequences of energy failure is thus essential for tackling bioenergetic diseases and requires quantitative analysis of the cellular ATP level. ATP is produced both by glycolysis in the cytoplasm and by OXPHOS in the mitochondria. However, little evidence demonstrates the consequences of dysregulated ATP levels in cells and their direct relevance in mitochondrial diseases. Needless to say, we lack a clear picture of how dysregulated ATP levels bridge between genotypes and mitochondrial diseases. Advancements in this area of research may provide new therapeutic tools for intervention with relevant diseases.

## Seeing is believing: Visualizing ATP signal at the single-cell level

Genetically encoded biosensors have enabled in vivo detection of cellular metabolites including ATP [12–15], NAD+/NADH [16], and lactate [17]. Compared to measuring metabolites in bulk lysates, genetically encoded fluorescent biosensors can provide insights into the temporal and subcellular dynamics of cellular metabolism in individual cells.

In 2009, two studies presented a new generation of ATP biosensors [13, 14]. In the first paper by Berg and colleagues, a circularly permuted green fluorescent protein (cpGFP) was combined with a bacterial ATP-binding protein to generate a reporter that quantitatively reports cellular ATP to adenosine diphosphate (ADP) ratio [14]. Because of differential binding affinities to ATP and ADP, the reporter exhibits different degrees of conformational change in cpGFP and thus different fluorescent signal changes in response to ATP or ADP binding. In the second paper, Imamura and colleagues developed a series of highly sensitive and selective Förster resonance energy transfer (FRET)-based reporters for cellular ATP concentrations. FRET-based reporters contain a subunit of a bacterial ATP synthase, fused between cyan fluorescent protein (CFP) and yellow fluorescent protein (YFP) [13]. The reversible binding of ATP leads to conformational changes of the synthase subunit, resulting in altered spatial proximities between fluorescent proteins and thus changes in the FRET signal. FRET-based reporters were modified to have different binding affinities to ATP, allowing measurements for a wide range of ATP concentrations. Based on these two types of ATP biosensors, a series of improvements and modifications have been made, including reporters with enhanced dynamic range [18] and excitation-free bioluminescence resonance energy transfer (BRET)-based reporters for whole-body imaging [19].

Single-cell studies using ATP biosensors are starting to reveal the regulation and function of ATP level in diverse areas of biology. Here, we briefly mentioned a few notable examples. In cancer biology, a major question regards how cancer cells take advantage of metabolic plasticity to regulate key functions such as growth and metastatic invasion. Studies with ATP biosensors reveal that under different growth perturbations, cancer cells can adjust cellular ATP levels by regulating glycolysis and OXPHOS in a synergistic manner [13], and a small fraction of tumor cells up-regulates ATP level (possibly by up-regulating OXPHOS via overexpressing H3K4-demethylase) and exhibits a slow-growing, multidrug-resistant, and invasive phenotype [20]. In developmental biology, the applications of ATP biosensors in oocyte maturation researches help to reveal how dynamic organizations of mitochondria in maturing oocytes can result in bursting ATP signals. These ATP signals may be critical for ensuring full developmental potential [21]. In neurobiology, studies using FRET biosensors for ATP demonstrated the essential roles of mitochondrial ATP in synaptic vesicle recycling, suggesting that energy failure may play key roles in neurodegeneration [11]. In cell biology, the study of the yeast cell cycle using an ATP biosensor has led to the discovery of unexpectedly oscillatory ATP signals on the cell-cycle timescale. Such oscillatory ATP signals are thought to be the result of the metabolic dynamic network and are not caused by the cell cycle [22]. How such dynamic ATP signals can affect downstream functions remains unknown. Although similar single-cell measurements have not yet been done in mammalian cells, existing population-level data suggest many metabolites, including ATP, appear to oscillate as well [23].

## ATP biosensors leap ahead: Integration with CRISPRi screen

**Fig 2:**
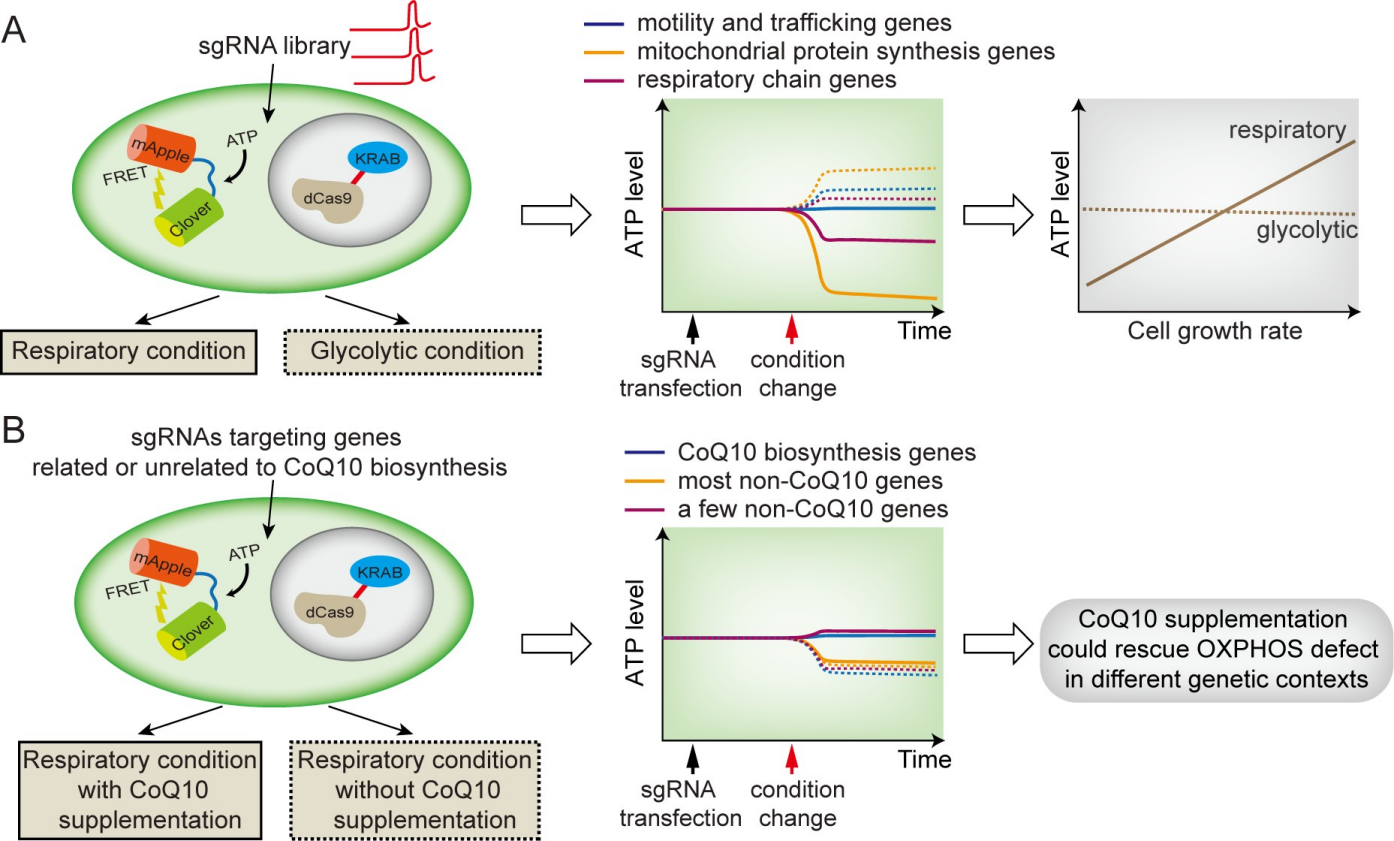
Combining real-time ATP biosensor with CRISPRi enables a single-cell–based high-throughput screening assay. (A) Assay design and results (schematics) [24]. (Left) Individual genes targeted by the sgRNA library were knocked down in cells expressing the improved ATP biosensor. Types of conditions are indicated by solid or dashed lines. (Middle) Gene knockdowns often result in opposite responses in cellular ATP levels for the respiratory versus the glycolytic condition. Experimentally, sgRNA-transfected cells were subjected to two different conditions, and the resulting effects on ATP level of different gene knockdowns were quantified. Here, types of genes are indicated by line colors, and solid or dashed lines indicate corresponding conditions on the left panel. (Right) The altered ATP levels affect cell-growth rates in a metabolic-context–dependent manner. Note that the ATP levels were not quantified in real time in the actual experiments. (B) The effects of CoQ10 supplementation (schematics) [24]. (Left) Screen design and screen conditions. Types of conditions are indicated by solid or dashed lines. (Middle) High CoQ10 supplementation can rescue respiratory ATP levels for both deficiencies in CoQ10 biosynthesis genes and a few non-CoQ10 biosynthesis-related genes. (Right) These results show that CoQ10 supplementation could rescue diverse mitochondrial respiration defects, suggesting its potential therapeutic applications in mitochondrial disorders. CoQ10, coenzyme Q10; CRISPR, clustered regularly interspaced short palindromic repeats; CRISPRi, CRISPR interference; OXPHOS, oxidative phosphorylation; sgRNA, single guide RNA.

Despite the improvements in ATP biosensors, most single-cell work utilizing these sensors has been limited by throughput because of the difficulty of studying single cells in high throughput. In a recent publication of *PLOS Biology*, Mendelson and colleagues overcame this limitation by combining a CRISPR interference (CRISPRi) screen with an improved ATP biosensor [24]. In this improved sensor, the CFP/YFP FRET pair is replaced with Clover/mApple, allowing accurate determinations of ATP levels in a high-throughput manner using standard flow cytometry lasers. In order to uncover key genes or pathways for bioenergetics and associated cellular functions, the authors characterized how individual gene knockdowns affect cellular ATP levels in a condition-specific manner and studied whether dysregulated ATP levels lead to growth phenotypes. To do so, they performed a CRISPRi screen at a subgenome level (a total of 2,231 genes with 10 guide RNAs per gene). This screen was done for three metabolic contexts (respiratory or OXPHOS only, glycolytic, and basal) (**Fig 2A**). Importantly, control experiments with a mutated ATP-sensor were included in all contexts, allowing the removal of non-ATP–dependent effects.

Based on the screen, the authors uncovered many genes that play major roles in cellular ATP level maintenance. Most of these genes are protein subunits of mitochondrial ribosomes and proteins involved in mitochondrial genome transcription and translation. Interestingly, genes related to mitochondrial protein synthesis show a more severe effect on ATP level than genes coding individual respiratory-chain components. This result suggests that combined mitochondrial defects (caused by deficiencies in mitochondrial protein synthesis) result in more severe energy failure than the dysfunctions of single respiratory-chain–complex subunits (which could be compensated by unknown mechanisms). In addition, the authors identified new genes unrelated to mitochondria function but reduce ATP production from mitochondria. These genes may modify cellular ATP level by influencing ATP consumption, suggesting the complex feedback mechanisms underlying ATP regulation. Furthermore, their data reveal metabolic-context–dependent ATP level regulation (**Fig 2A**). Specifically, some mitochondrial gene deficiencies decrease cellular ATP level only in respiratory conditions but not in conditions when glycolysis is engaged, indicating that glycolysis may compensate for the decreases in ATP when OXPHOS is perturbed. This result highlights the plasticity of metabolic systems as well as the challenges for linking genotypes to disease phenotypes. This may further explain the lack of direct evidence connecting dysregulated ATP levels to mitochondrial diseases. Therefore, the results by Mendelson and colleagues call for more attention to context-dependent ATP level regulation when studying energy failure.

To further explore how additional environmental factors regulate cellular ATP levels, the authors characterized the effect of supplementing the culture media with coenzyme Q10 (CoQ10), a widely available dietary supplement for treating mitochondrial disorders without clear mechanisms [25]. Interestingly, high-level supplementation of CoQ10 to cells lead to increased ATP levels in cells with diverse genetic mutations, a few of which are not even involved in CoQ10 biosynthesis. This result suggests the broad effect of CoQ10 on mitochondrial functions and provides a new hint for disease therapies related to bioenergetics (**Fig 2B**).

To dissect how dysregulated cellular ATP levels affect downstream phenotypes such as growth rates, the authors analyzed growth rates of cells under different genetic and environmental perturbations. They found that ATP dysregulation affects cell growth rates in a metabolic-context–dependent manner (**Fig 2A**). More specifically, for mitochondrial-related genes whose deficiencies reduce ATP levels, their deficiencies only impact growth when OXPHOS is active. Based on these observations, the authors proposed an energy-based therapeutic approach to eliminate cancer cells by exploiting context-specific growth inhibition of cancer cells that have certain gene mutations (that affect cell growth in a context-dependent manner).

## Summary and outlook

The study by Mendelsohn and colleagues demonstrates how quantifying cellular ATP concentration at the single-cell level in a high-throughput manner help to establish links between genotypes and cellular phenotypes or diseases. For the identified genes that are linked to particular phenotypes, it will be of great interest to investigate whether and how temporal and spatial ATP level dynamics play roles in these phenotypes. This would require the development of live-cell assays for monitoring ATP signals and the downstream processes simultaneously in the same cells.

The screening methodology developed by Mendelsohn and colleagues could be extended to additional gene libraries and address how other genetic pathways may contribute to cellular ATP level regulation. It can also be used for the screening of metabolic substrates and/or drug combinations as candidate therapies for energy failure. To arrive at a systems-level understanding of mitochondrial biology and metabolism in general, similar single-cell–based screening approaches can be adapted to other metabolic products (e.g., NADH [16], lactate [17], and reactive oxygen species (ROS) [26]). Understanding how different metabolites are regulated at a systems level may shed light on distinguishing mitochondrial diseases originating from disorders in the regulation of different metabolites.

More generally, single-cell approaches open up new opportunities for understanding the causes and consequences of cell-to-cell variability in metabolites. In both bacterial and mammalian cells, it has been reported that ATP levels can display a large cell-to-cell variability even in a clonal population [27, 28]. While it has been suggested that mitochondrial dynamics may be responsible for such variabilities in mammalian systems [28], complex gene regulatory or metabolic networks may also play some roles in the measured variabilities. Given the significant roles that ATP plays in diverse biological processes, it is particularly important to study how the variability or dynamics of ATP signals affect biological functions at the cellular, tissue, and organismal level.

## Abbreviations

ADP: adenosine diphosphate
AMP: adenosine monophosphate
BRET: bioluminescence resonance energy transfer
CFP: cyan fluorescent protein
CoA: coenzyme A
CoQ10: coenzyme Q10
cpGFP: circularly permuted GFP
CRISPR: clustered regularly interspaced short palindromic repeats
CRISPRi: CRISPR interference
ETC: electron transport chain
FRET: Förster resonance energy transfer
GFP: green fluorescent protein
OXPHOS: oxidative phosphorylation
ROS: reactive oxygen species
sgRNA: single guide RNA
TCA: tricarboxylic acid
YFP: yellow fluorescent protein
